# Certificated Blind Masseurs

**Published:** 1920-11-20

**Authors:** Bruce Bruce-Porter


*To the Editor of* THE HOSPITAL.

### Certificated Blind Masseurs.

*To the Editor of* THE HOSPITAL.

SIR,—I have read with interest the letter written by Colonel Mansell Moullin with reference to the Blind Masseurs. I had considerable experience of the work of these men when in command of the Third London General Hospital during the war, and very cordially support all that has been written by Colonel Mansell Moullin. The whole question of massage is a very serious one for the medical profession, as we are held responsible for the results of the treatment we recommend, and, when, as there are so many trained and certificated masseurs and masseuses in the country as there are to-day, it is our duty towards our patients to make sure that, when we prescribe massage it is carried out by trained workers, who have obtained the certificates of recognised training societies, such as the Incorporated Society of Trained Masseurs (since reconstructed as the Chartered Society of Massage and Medical Gymnastics) and not by people who have had no training, or who are merely acquiring practice without having undergone a thorough training.

May I accentuate the fact that all members of the Association of Certificated Blind Masseurs, referred to by Colonel Mansell Moullin, have been thoroughly trained and hold recognised qualifications? Finally, too much stress cannot be laid upon the fact that the members of the Association only undertake the treatment of patients with the consent and advice of a registered medical practitioner.—Yours faithfully,

BRUCE BRUCE-PORTER.

